# Murine Hepatitis Virus Exoribonuclease nsp14 Is Required for the Biogenesis of Viral Circular RNAs

**DOI:** 10.1128/spectrum.04460-22

**Published:** 2023-05-15

**Authors:** Shaomin Yang, Ruth Cruz-Cosme, Di Cao, Hong Zhou, Songbin Wu, Jiabin Huang, Zhen Luo, Hua Zhu, Qiyi Tang

**Affiliations:** a Department of Pain Medicine and Shenzhen Municipal Key Laboratory for Pain Medicine, Huazhong University of Science and Technology Union Shenzhen Hospital, Shenzhen, China; b Howard University College of Medicine, Washington, DC, USA; c Shenzhen Nanshan People’s Hospital of Shenzhen University Health Science Center, Shenzhen, China; d Rutgers University, Newark, New Jersey, USA; Wayne State University

**Keywords:** murine hepatitis virus, nsp14, circRNA, RNA splice junction

## LETTER

Circular RNA (circRNA), a newly identified important component of the transcriptome, is formed by covalently bonded single-stranded RNA through back splicing (1) or other unknown mechanisms. It has been reported that circRNA plays important biological functions including microRNA (miRNA) sponges, parental gene expression regulators, and the translation template (2–5). Viral circRNAs have been recently identified from cells infected with different DNA viruses, such as Epstein-Barr virus (EBV) (6, 7), Kaposi’s sarcoma-associated herpesvirus (KSHV) (8), human papillomaviruses (HPVs) (9), and human cytomegalovirus (HCMV) (10). Coronavirus disease 2019 (COVID-19), caused by the severe acute respiratory syndrome coronavirus 2 (SARS-CoV-2), has become a worldwide pandemic and poses a high threat to global health. We unprecedentedly identified viral circRNAs from cells that were infected by different coronaviruses, including SARS-CoV-2, SARS-CoV, and Middle East respiratory syndrome (MERS)-CoV (11). However, the biogenesis of circRNAs from coronavirus is still unknown.

Murine hepatitis virus (MHV), a betacoronavirus, has been used in a mouse model to study human coronaviruses (12). Gribble et al. (13) reported that an RNA proofreading exoribonuclease, nsp14-ExoN, encoded by the MHV genome contributes to RNA recombination. In their study, deep transcriptome sequencing (RNA-seq) was performed on murine DBT cells infected either with wild-type MHV (MHV-WT) or with nsp14-ExoN inactive mutant MHV (MHV-ExoN^−^) (Table 1). We hypothesized that nsp14-ExoN may mediate the biogenesis of MHV circRNAs. To systematically test this hypothesis, we analyzed RNA from MHV-WT- or MHV-ExoN^−^-infected cells and virion-containing supernatants. Currently used methods of identifying circRNA are established on the examination of the back-splicing junction (BSJ) (14).

**TABLE 1:** Information on the RNA-seq data set used in this study^*a*^

NCBI SRA ID	Virus	RNA source	Genome coverage	Sum		Frequency	
				BSJs	FSJs	BSJs	FSJs
SRR11486440	MHV-WT	Cell monolayers	34,449,280	370,579	984,698	107.5723	285.8399
SRR11486441	MHV-WT	Cell monolayers	34,355,466	381,676	995,163	111.0961	289.6666
SRR11486442	MHV-WT	Cell monolayers	33,593,523	408,580	1,038,705	121.6246	309.198
SRR13389004	MHV-ExoN^−^	Cell monolayers	48,586,710	91,854	239,967	18.90517	49.38943
SRR13389005	MHV-ExoN^−^	Cell monolayers	44,450,534	84,030	207,637	18.90416	46.71192
SRR13389006	MHV-ExoN^−^	Cell monolayers	42,623,617	77,786	210,224	18.24951	49.32101
SRR11486224	MHV-WT	Viral supernatant (virion)	50,795,034	81,980	204,536	16.13937	40.26693
SRR11486225	MHV-WT	Viral supernatant (virion)	56,476,922	97,276	297,169	17.22403	52.61778
SRR11486226	MHV-WT	Viral supernatant (virion)	55,943,977	97,867	191,965	17.49375	34.31379
SRR11486433	MHV-ExoN^−^	Viral supernatant (virion)	47,259,994	51,616	100,737	10.92171	21.31549
SRR11486434	MHV-ExoN^−^	Viral supernatant (virion)	32,558,489	52,430	74,919	16.10333	23.01059
SRR11486435	MHV-ExoN^−^	Viral supernatant (virion)	23,043,062	37,353	75,753	16.21009	32.87454

a MHV reference genome, NC_048217.1. ID, identifier.

**FIG 1:**
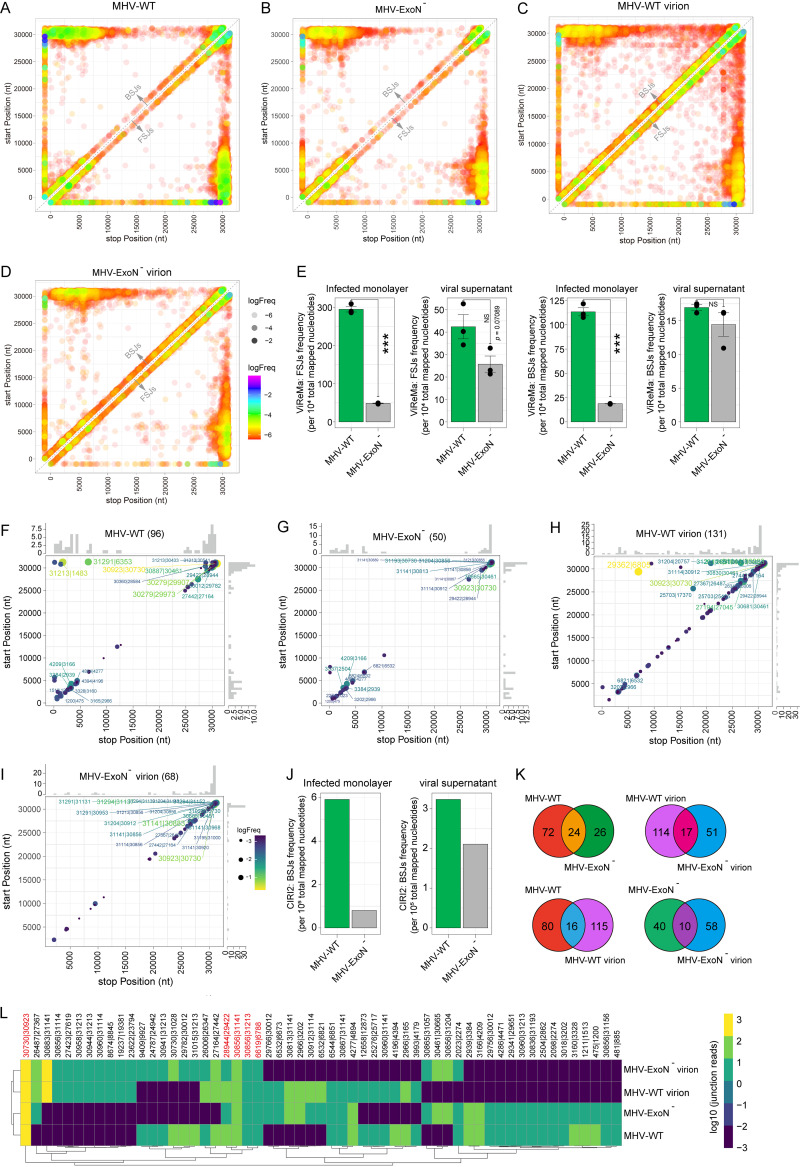
nsp14-ExoN mediated the biogenesis of circRNAs in MHV-infected cells. (A to D) Forward-splicing junction and back-splicing junction events in cells infected with MHV-WT (A), MHV-ExoN^−^ (B), MHV-WT virion (C), or MHV-ExoN^−^ virion (D). nt, nucleotide. MHV reference genome: NC_048217.1. (E) Statistics of the junction frequency. Statistical significance was determined by the unpaired Student *t* test. NS, not significant; ***, *P* < 0.0001. *n* = 3 per group. Data are presented as mean ± standard error of the mean. (F to I) Frequency of circularization events in cells infected with MHV-WT (F), MHV-ExoN^−^ (G), MHV-WT virion (H), and MHV-ExoN^−^ virion (I). Counts of BSJ-spanning reads (starting from a coordinate on the *x* axis and ending in a coordinate on the *y* axis) indicated by color and point size. The counts were aggregated into 500-nucleotide bins for both axes. The distributions of start/end positions are shown as histograms on the *x* and *y* axes. (J) Statistics of MHV circRNA frequency. (K) Venn diagram presenting the number of unique or shared circRNAs in each paired group. (L) Heatmap of the shared circRNAs from at least two groups.

ViReMa is one such tool that can quickly and sensitively identify viral RNA splicing junctions, including forward-splicing junctions (FSJs) and BSJs from next-generation sequencing data (15). Therefore, we applied ViReMa to assess the abundance of MHV-derived BSJs and FSJs and mapped the breakpoints to their respective genomic locations (Fig. 1A to D). In summary, there are two significant hot spots of FSJs and BSJs: (i) distant splicing between the 3′ and the 5′ ends of the genome corresponding to the N gene and the untranslated region (UTR) and (ii) local splicing in regions of MHV. The statistical analysis of genome coverage shows that about 23,043,062 to 56,476,922 nucleotides of each sample were mapped to the MHV genome (Table 1), suggesting that the numbers of MHV RNA molecules in the samples are comparable. To assess the effect of nsp14-ExoN loss of function, we calculated the junction frequency by normalizing to the genome coverage, which has been described by Gribble et al. (13). The results of the statistical analysis show that the loss of MHV nsp14-ExoN led to significant decreases in BSJ and FSJ frequencies in infected cells but not in the viral supernatant (Fig. 1E). These data indicate that nsp14-ExoN may mediate the formation of both FSJs and BSJs of MHV.

In our previous study, we found that CIRI2, as a circRNA prediction pipeline, is more reliable for coronavirus circRNA identification than other RNA prediction algorithms (11). Therefore, we also applied CIRI2 to identify MHV circRNAs. To improve the identification accuracy, biological replicates of the same condition were pooled. As shown in Fig. 1F to I, the CIRI2 pipeline identified 96, 50, 131, and 68 MHV circRNAs in the MHV-WT- and MHV-ExoN^−^-infected cell monolayers and and MHV-WT and MHV-ExoN^−^ viral supernatant, respectively. By normalizing the genome coverage, the number of MHV circRNAs in MHV-ExoN^−^-infected cells (Fig. 1J) is significantly lower than that in MHV-WT-infected cells. These results indicate that nsp14-ExoN is required for the biogenesis of MHV circRNAs. Interestingly, we noticed that most of the circRNAs of MHV-WT- and MHV-ExoN^−^- or MHV-infected cell monolayers and viral supernatants were different (Fig. 1K). Only 5 circRNAs were found in both MHV-WT- or MHV-ExoN^−^-infected cells and the supernatant (Fig. 1L, red). These data suggested that nsp14-ExoN may serve as a viral proof factor to correct the breakpoint of MHV BSJs.

**FIG 2:**
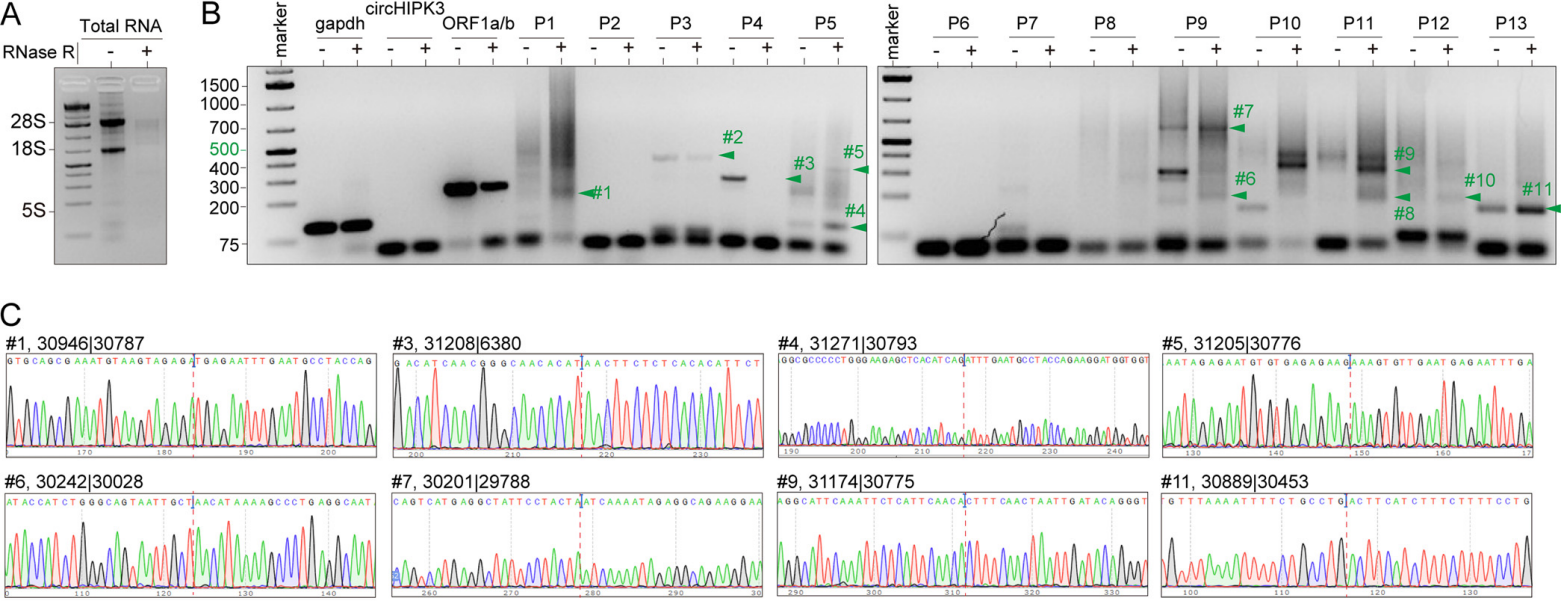
Experimental identification of MHV circRNAs. (A) An agarose gel analysis of total RNA with and without RNase R treatment. (B) Inverse reverse transcription-PCR (RT-PCR) result of the MHV with divergent primers. Products of RT-PCR from the total RNA that was untreated or treated with RNase R. Bands indicated by arrowheads were TA cloned and sequenced. (C) Representative Sanger sequencing results are shown. BSJ breakpoints are indicated by dashed lines. Mouse linear RNA, glyceraldehyde-3-phosphate dehydrogenase (GAPDH), MHV linear RNA, ORF1ab, mouse circRNA, circHIPK3, and MHV circRNAs.

To experimentally confirm viral circRNAs from MHV-infected cells, we extracted total RNA from murine DBT cells that were infected with the MHV-A59 strain at 48 h postinfection (hpi). circRNAs have a covalently closed configuration and are hence more resistant to exoribonuclease RNase R than linear RNAs (16). Thus, we treated total RNA with RNase R for 30 min, and agarose gel electrophoresis showed that most rRNAs were degraded after RNase R treatment (Fig. 2A). Divergent primers were designed to amplify the targeted BSJs of the most abundant MHV-encoded circRNAs in the region of 29,000 to 31,300 (see Data Set S1 in the supplemental material). To determine whether the inverse PCR products were from BSJs rather than nonspecific PCR products, we gel purified candidate BSJ amplicons based on molecular weight (Fig. 2B) and performed subcloning and Sanger sequencing for each candidate (Fig. 2C and Data Set S1). Consequently, linear viral RNA derived from ORF1a/b was degraded by RNase R. In contrast, a well-characterized mouse circRNA circHIPK3, BSJ#1 (30946|30787), BSJ#4 (31271|30793), BSJ#7 (30201|29788), BSJ#9 (31174|30775), and BSJ#11 (30889|30453), still remained. These data confirmed that MHV-infected cells generate virus-specific circRNAs.

Our overarching findings of this study include that MHV encodes circRNAs and nsp14-ExoN is important for the biogenesis of MHV circRNAs. Since nsp14 is required for RNA recombination (13), we speculate that MHV circRNAs may participate in viral RNA recombination-mediated mutations, which certainly needs to be further demonstrated. Thus far, circRNAs have been identified from several human DNA and RNA viruses. It has been reported that cellular circRNAs significantly impact the replication of MERS-CoV (17) and Bombyx mori nucleopolyhedrovirus (18). However, the biological functions of viral circRNAs largely remain unclear. The studies presented here may open a new avenue to explore the roles of viral circRNAs in viral pathogenesis, evolution, and replication. Due to the high stability of circRNAs, they may be involved in the long-term pathogenic effects of a viral infection such as long COVID. Further research on human coronaviruses such as SARS-CoV-2, SARS-CoV, and MERS-CoV should be conducted to better understand the function of viral circRNAs and the role played by nsp14-ExoN.

### Data availability.

Extended data are available from Mendeley Data: https://data.mendeley.com/datasets/kw453xnjkh/draft?a=c3c9ee47-38f6-43f3-84bd-41b4d2378281.
