# Supplementary material for: Murine Hepatitis Virus Exoribonuclease nsp14 Is Required for the Biogenesis of Viral Circular RNAs
Source: Microbiol Spectr. 2023 May 15;11(3):e04460-22. doi: 10.1128/spectrum.04460-22 (PMC10269776; doi:10.1128/spectrum.04460-22)
Supplement: Supplemental file 1 — Data Set S1. Download spectrum.04460-22-s0001.docx, DOCX file, 0.02 MB [file spectrum.04460-22-s0001.docx]

**Primers used in this study:**

| Primer set name | Primer name | Sequence (5' to 3') |
| --- | --- | --- |
| gapdh | Mouse gapdh-PF | AAATGGTGAAGGTCGGTGTG |
|  | Mouse gapdh-PR | TGAAGGGGTCGTTGATGG |
| ORF1ab | ORF1ab-PF | TGAAGGCATTGTGCGTGTTG |
|  | ORF1ab-PR | ACTCGCCGTTAAGGCAAAGA |
| circHIPK3 | Mouse circHIPK3-PF | GGATCGGCCAGTCATGTATC |
|  | Mouse circHIPK3-PR | ACCGCTTGGCTCTACTTTGA |
| P1 | 30730-30923-PF | CCCCAAAGAAAAGGGCGTAGA |
|  | 30730-30923-PR | CCATCCTTCTGGTAGGCATTCA |
| P2 | 6805-29362-P1F | GTTCGGTTACACGAGCCGTA |
|  | 6805-29362-P1R | TCAGGCAACCATGTGGTCAA |
| P3 | 1483-31213-P1F | TGAATCCTATGTCGGCGCTC |
|  | 1483-31213-P1R | TTCTGACAACGGCTACACCC |
| P4 | 6353-31291-P1F | TGAATCCTATGTCGGCGCTC |
|  | 6353-31291-P1R | CACTAGCAGAAGCACAGGCT |
| P5 | 28000\|31330-P1F | AGGTTGTGGCAGACCCTGTA |
|  | 28000\|31330-P1R | GAGCCTGTCTACGCCCTTTT |
| P6 | 6805\|29362_F | CTTTGGTGCTGTCCCCTTCT |
|  | 6805\|29362_R | CCACGCAACTATCTGTCCCA |
| P7 | 1483\|31213_F | AAAAGGGCGTAGACAGGCTC |
|  | 1483\|31213_R | TGCTCGAGATTGCCACACTT |
| P8 | 29907\|30279_F | AGCCAAGCGGACACCAATAC |
|  | 29907\|30279_R | TCCCTTTTGCTCTGAAGCGG |
| P9 | 29973\|30279_F | GTCGAAAGGGACCCAAGCAG |
|  | 29973\|30279_R | GGCCCTGTGCCAAGATAGTA |
| P10 | 30883\|31141_F | TGAATCCTATGTCGGCGCTC |
|  | 30883\|31141_R | TCTGGCACTACGCCATCATC |
| P11 | 30965\|31114_F | GGTAACCCCTCGCGAGAAAG |
|  | 30965\|31114_R | TAACCCATCTGGCACTACGC |
| P12 | 30461\|30887_F | ATGGTGGTGCAGATGTGGTG |
|  | 30461\|30887_R | GTCCTCTTTTGGCGAGGCTT |
| P13 | 30461\|30665_F | CCACAGTTCCCCATTCTTGC |
|  | 30461\|30665_R | CACTGGGCACTGCTTGTTTG |

**Experimentally confirmed MHV circRNAs:**

| CircRNA | Start | End | Length | Primer name |
| --- | --- | --- | --- | --- |
| 1 | 30787 | 30946 | 159 | P1 |
| 2 | 29885 | 30201 | 316 | P9 |
| 3 | 6380 | 31208 | 24828 | P4 |
| 4 | 30793 | 31271 | 478 | P5 |
| 5 | 30776 | 31205 | 429 | P5 |
| 6 | 30028 | 30242 | 214 | P9 |
| 7 | 29788 | 30201 | 413 | P9 |
| 8 | 30424 | 30726 | 302 | P11 |
| 9 | 30775 | 31174 | 399 | P11 |
| 10 | 30739 | 30990 | 251 | P12 |
| 11 | 30453 | 30889 | 436 | P13 |
| 12 | 30781 | 30993 | 212 | P5 |
| 13 | 30784 | 30924 | 140 | P5 |
| 14 | 30843 | 30924 | 81 | P11 |
| 15 | 30880 | 31113 | 233 | P11 |
| 16 | 29894 | 30201 | 307 | P9 |
